# Physics Clues on the Mind Substrate and Attributes

**DOI:** 10.3389/fncom.2022.836532

**Published:** 2022-04-08

**Authors:** Joaquin J. Torres, Joaquín Marro

**Affiliations:** Institute Carlos I for Theoretical and Computational Physics, University of Granada, Granada, Spain

**Keywords:** collective brain phenomena, adaptive complex networks, dynamic synapses, non-equilibrium phase transitions, EEG oscillations, intelligence, identity, consciousness

## Abstract

The last decade has witnessed a remarkable progress in our understanding of the brain. This has mainly been based on the scrutiny and modeling of the transmission of activity among neurons across lively synapses. A main conclusion, thus far, is that essential features of the mind rely on collective phenomena that emerge from a willful interaction of many neurons that, mediating other cells, form a complex network whose details keep constantly adapting to their activity and surroundings. In parallel, theoretical and computational studies developed to understand many natural and artificial complex systems, which have truthfully explained their amazing emergent features and precise the role of the interaction dynamics and other conditions behind the different collective phenomena they happen to display. Focusing on promising ideas that arise when comparing these neurobiology and physics studies, the present perspective article shortly reviews such fascinating scenarios looking for clues about how high-level cognitive processes such as consciousness, intelligence, and identity can emerge. We, thus, show that basic concepts of physics, such as *dynamical phases* and *non-equilibrium phase transitions*, become quite relevant to the brain activity while determined by factors at the subcellular, cellular, and network levels. We also show how these transitions depend on details of the processing mechanism of stimuli in a noisy background and, most important, that one may detect them in familiar electroencephalogram (EEG) recordings. Thus, we associate the existence of such phases, which reveal a brain operating at (non-equilibrium) criticality, with the emergence of most interesting phenomena during memory tasks.

## Introduction

As humans we are interested in the age-old question *What are we?*, perhaps now rephrased *Can one identify guidelines to understand our intimate being?* The doubt is not banal. Looking into this requires involving the mind that, for a very long time, has been an ambiguous entity, and therefore source of misunderstandings and unfortunate hypotheses. However, by developing new means of observation and computation, science has uncovered details, and paths are now open on which to begin to walk confidently. So much so that we may rationally precise, for instance: *What makes us be the way we are*? *Where is located our own identity*? *Could it be manipulated*? Even more, it has been uncovered that probing solutions to these queries equals looking for the keys of our *identity* and *consciousness* and, in trying to do so, it has been realized that the relevant scenarios closely relate to *intelligence*.

According to thesauruses, intelligence is “ability to acquire and apply knowledge and skills,” and relate this term to “understand,” “solve problems,” “experience,” and “competence.” We stuck to this. Nevertheless, we are not interested in meanings such as “purely spiritual substance,” and forget for the moment the so-called *social intelligence*—the one ascribed to *groups* of ants, bees, birds, fish, and humans. In any case, this eclectic vision is insufficient for us, as it hides the essence or content of what we could name intelligence “function,” i.e., how those capacities unfold as an essential part of our being. This is what most interests us here.

We manage to go one-step farther by exploring what in this connection distinguishes the animal species. Perhaps it then surprises that the sperm whale has a bigger brain than we have, and that the shrew's one weights more relative to the total. To have humans leading a list in this context, we must ponder the neurons connectivity—in which case however dolphins follow us, not primates as one might have expected (Roth and Dicke, 2005). Endorsing a popular identification, this associates intelligence with *gray matter* that, leaving aside other structures, consists of near 100 billion *somas* or neural bodies. These extend through long eager-to-connect filamentous extensions that end in terminals with a complicated internal structure and one may call *synapses*, the name of one of its parts. All this stuff forms the cerebral *cortex* that, just under the skull, is the best-organized part of our very well-organized nervous system. The 10^11^ neurons (Azevedo et al., 2009) thus continually interact with any part of our body, including muscles, organs, and senses, through about 10^15^ synapses mainly in the neocortex (DeFelipe et al., 2002).

This picture suggests that properties of the cortex, such as its thickness, may be related and perhaps aid to estimate the intellectual capacity of an individual. However, today we understand this, together with some of the mechanisms that improve the brain operation and eventually may induce its malfunction, in more detail. For example, we have a harmonious framework (Marro, 2014; Marro and Torres, 2021), coherent with what we learned from experiments, which allows one to quantitatively exploring intriguing phenomena associated to the concept of intelligence and the mechanisms that seem to favor it up. This is a simple though rigorous scheme, kind of “mathematical metaphor” that includes, together with other details, a realistic description of synaptic cooperation between neurons. That is, it does not presume a passive participation of the synapses, but it specifies how these, constantly using both intrinsic and external information, actively modulate the interrelation between neurons, including its network effective topology, which affects, even dramatically, the current result of that collaboration. In short, synapses should now be viewed as effective processors responsible for achieving certain, fruitful, mutual influence between neurons at every time, and they do so with the mediation of several relatively complex biophysical mechanisms. Insomuch that these show up to the observer (using suitable techniques) as “noises” or fluctuations along several time scales propagating through an adapting complex network. These “noises” carry relevant information that characterizes some brain activity states (Lendner et al, 2020), and may be important to understand brain interrelations (Waschke et al., 2021), so that the resultant scene is very subtle.

This perspective article shortly reviews the framework supporting the above scenarios, namely, we follow here a statistical physics point of view looking for indications about how high-level cognitive processes such as consciousness, intelligence, and identity can emerge. In particular, we, thus, illustrate how basic concepts of physics, such as *dynamical phases* and *non-equilibrium phase transitions*, are quite relevant for the emergence of intriguing synchronization phenomena and for the understanding of the dynamical features of actual brain activity which can be related with different brain cognitive functions. In addition, we explore the factors at the subcellular, cellular, and network levels that seem to induce the non-equilibrium phases that happen to show up, and remark the important role that synaptic mechanisms and network development, and refinement processes such as synaptic pruning, have on the observed phenomena. We also show here that the nature of the relevant non-equilibrium transitions depends on how incoming stimuli are processed in a noisy background, which might provide a useful and plausible tool to detect them in actual electroencephalogram (EEG) recordings. Additionally, we, thus, associate the existence of such phases, which reveal a brain operating at (non-equilibrium) criticality, with the emergence of the most interesting phenomena during memory tasks.

## A First Mesoscopic View

An important and widely accepted fact here is that the synaptic actions connecting neurons are conditioned by “memories,” namely, patterns that previously stored within during a kind of *learning process* and which are constantly adapted throughout the individual life due to new acquired information (Hebb, 1949; Amit, 1989). That is, by means of biophysical processes, we plastically store pieces of information (sensory perceptions, behavioral procedures, etc.) in our synapses, and we continually update that data while undergoing new circumstances (Marro and Torres, 2021). In practice, this happens to determine a very changing agenda of neuronal collaborations, which constantly conditions most high-level mental functions. On the other hand, in association to each mental process, there is now clear evidence that sets of synapses organize themselves into specific dynamics whose objective is to achieve constantly operating economy and proximity between different, even quite distant regions (Muñoz, 2018). Thus, by a proper combination of all this—mainly, continuous modulation of the synaptic interactions between neurons as well as eventual efficient coordination among groups of them—most elaborated mental properties emerge, including human intelligence and associated high-level functions such as working memory (Mongillo et al., 2008) or episodic memoires (Takeuchi et al., 2013). In particular, definite correlations between the familiar intelligence coefficient IQ and properties of the underlying neuron network, including its topology, effectiveness in transmitting information throughout, and the synaptic links dynamic activity have been reported (Li et al., 2009). This complex scenario is celebrated at the light of the relative components simplicity producing it.

The fact is that the mind functions and how the brain manages to structure itself just result from cooperation among (very many) rather humble neurons mediating continuous dynamic actions of their synaptic links, which may eventually (a few or many) abstain from acting (Marro and Torres, 2021). A crucial aspect of this image is that synaptic fluctuations, especially those on short time scales, are determined to induce and constantly maintain a situation that physics describes as “*critical*” (Muñoz, 2018), which is in all similar to the one that characterizes the so-called *critical points* in condensed matter phenomena such as, for instance, *condensation* and *ferromagnetism* (Stanley, 1987). This intriguing situation is characterized, for instance, by the appearance of *avalanche* dynamics for neural population activity showing power law distributions (Beggs and Plenz, 2003), as well as by the existence of long-range correlations in space and time that have recently been associated with the sense of identity (Sugimura et al., 2021). Therefore, neuroscientists have today the possibility to explore the emergence of such “critical” conditions during brain activity as many theoretical and experimental works, including neuronal cultures, functional MRI (fMRI) data, EEG time series, have already revealed (Beggs and Plenz, 2003; Tagliazucchi et al., 2012; Yaghoubi et al., 2018; Fontenele et al., 2019). This notion of criticality, brought to these non-equilibrium settings from the study of equilibrium physical systems, can be extended to the concept of the Griffiths phase (Griffiths, 1969) in the brain with structural heterogeneity. In fact, the existence of *critical* zones has been computationally verified for humans and *Caenorhabditis elegans* connectomes (Moretti and Muñoz, 2013). This clarifies our understanding of how some high-level cognitive functions can emerge during brain operation, as well as possible clinical applications to some neurological disorders (Zimmern, 2020). Furthermore, particular features of such emerging critical state can be important to understand our capacity to solve problems and make decisions, thus conforming our intelligence and identity (Ezaki et al., 2020; Jiang et al., 2021) as we explore next.

## Intelligence and Identity

**Figure 1:**
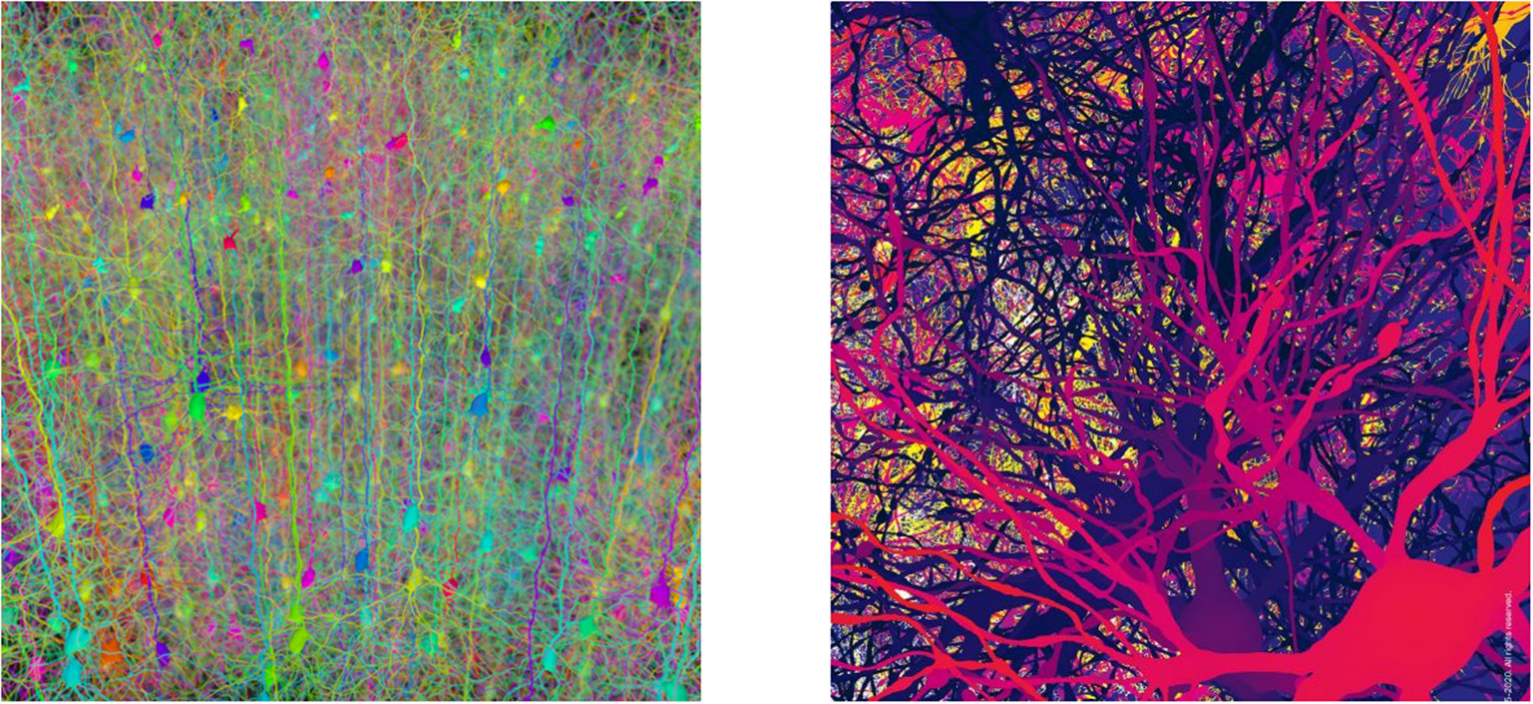
Forests of somas and synapses. The image on the left is a forest of synthetic pyramidal dendrites grown using Cajal's laws of neuronal branching (Image Credit: Hermann Cuntz, licensed under Creative Commons Attribution License, PLoS Comput Biol 6(8): ev06.i08. https://doi.org/10.1371/image.pcbi.v06.i08). The image on the right is a visualization of neurons in a digitally reconstructed thalamus model from the “Blue Brain Project” in École Polytechnique Fédérale de Lausanne (Image Credit: ©Blue Brain Project/EPFL 2005 – 2020. All rights reserved).

One may highlight now two main aspects that concern intelligence. Firstly that we, as humans, essentially are kind of mixture of neuron collaborations and time variations of the intensities at which synapses happen to relate them. Even popular newspapers long ago recognized that “*Brainpower May Lie in Complexity of Synapses*” (Wade, 2008), then properly explaining that “*synapses get considerably more complex going up the evolutionary scale […] It is likely this is one of the design principles by which the human brain is constructed*.” Indeed, it is sensible to say that our most important part as humans is the whole of our about 10^15^ synapses (DeFelipe et al., 2002). It is this mesh what likely houses our identity. In this way, each of us is uniquely—as a matter of probability—identified by information contained in all this enormous wired set of filaments (see Figure 1). This is the main of our identity, namely, all the data there plastically stored, which is a mixture of genetic inheritance and information frequently acquired and updated. Thanks to this immense and continually renewed data warehouse, the whole of processes that we associate with our intelligence are able, at any time and quickly, of remembering, combining, contrasting, and making decisions, computing, etc., making it possible what we call consciousness. Hence, the identity relying on this can diminish in any measure, due to loss or deterioration of part or that entire network or of the mechanisms that make it to correctly work and be efficiently useful, but we do not imagine how it could be transferred to another human being or exchanged with actual technology. In this sense, it is crucial understanding how the brain wiring network develops from conception until its mature form. In particular, this will help to understand the origin of brain network disorders, such as Autism Spectrum Disorder (Tang et al., 2014), schizophrenia (Keshavan et al., 1994), epilepsy (Andoh et al., 2019), and its Alzheimer deterioration.

**Figure 2:**
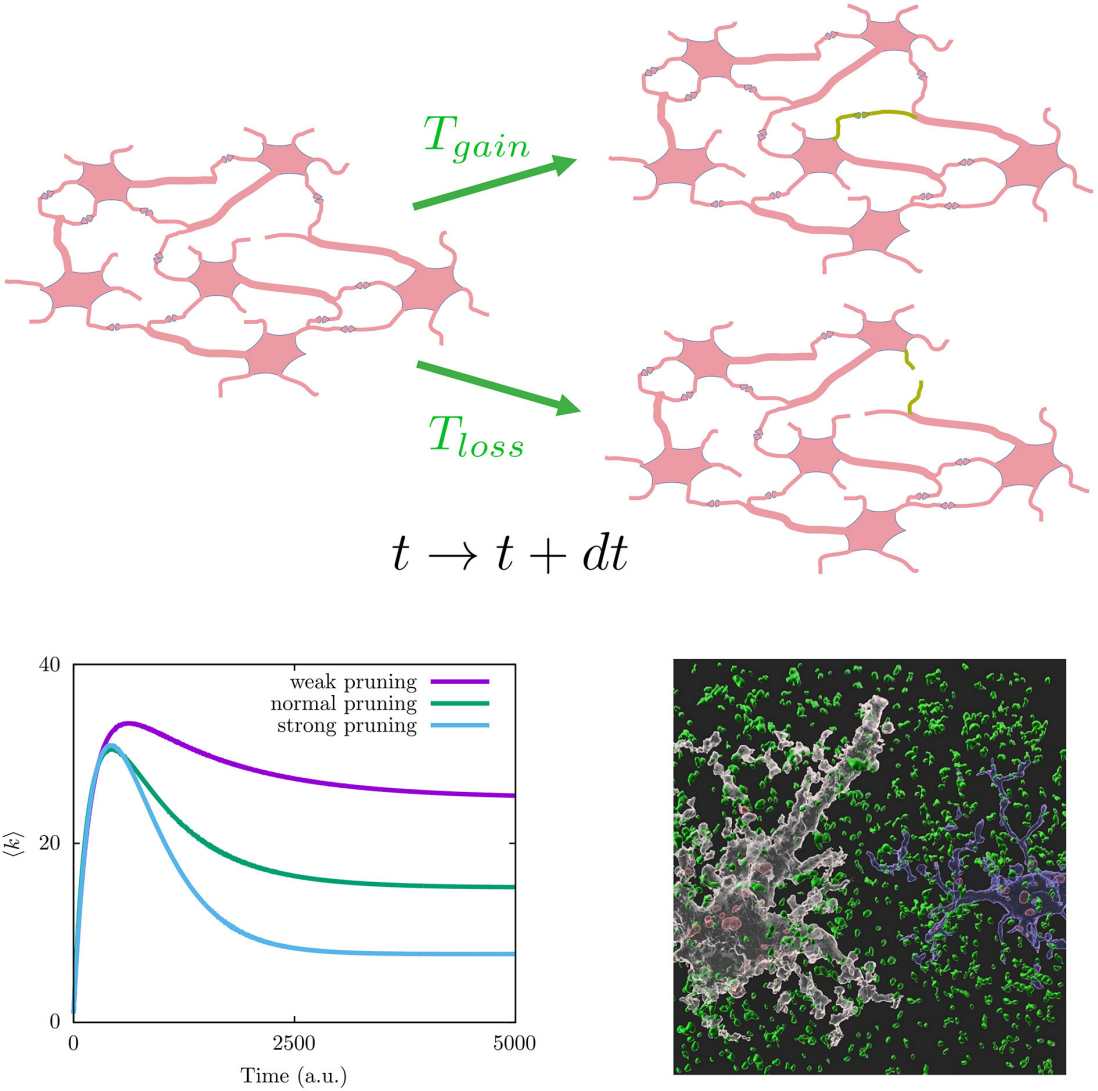
(Top) Graph interpretation of the mathematical model in Equation (1). Given a neuronal network configuration at time *t* (left), a new synapse can be generated (lost) with probability *T*_*gain*_(*T*_*loss*_) at time *t*+*dt* (*green connections on the right neuronal network configurations*). (Bottom left) Different synaptic pruning curves produced by Equation (1) (details for particular choices for transition probabilities *T*_*gain*_ *and T*_*loss*_ can be found in Johnson et al., 2010). (Bottom right) Image showing synapse phagocytosis by astrocytes reported in mouse hippocampus (Lee et al., 2021). Presynapses are colored in green, astrocytes in white, and microglia in blue. Phagocytosed presynapses by glia were shown in red. Image has been taken from the Korea Advanced Institute of Science and Technology (KAIST) (Image credit: Won-Suk Chung, https://www.kaistglia.org/).

In fact, understanding dynamical principles of how our brain structure develops has attracted some attention (Tetzlaff et al., 2010; Millán et al., 2018, 2019, 2021). It was reported, for example, that a suitable mathematical framework to study brain development is the master equation for the neuron degree probability distribution *p*(*k*) (Johnson et al., 2009, 2010; Millán et al., 2018):


(1) $$\begin{array}{l} \frac{dp\left(k,t\right)}{dt}=T_{gain}\left(\kappa ,\;k-1,\ldots \right)p\left(k-1,t\right)+T_{loss}\left(\kappa ,\;k+1,\ldots \right)\\ p\left(k+1,t\right)-\left[T_{gain}\left(\kappa ,\;k,\ldots \right)+T_{loss}\left(\kappa ,\;k,\ldots \right)\right]p\left(k,t\right) \end{array}$$

where it is considered different type of microscopic mechanisms to add and remove synapses with time, here represented, respectively, by the transition rates *T*_*gain*_(κ, *k*, …) to increase the number of neighbors of a given neuron from *k to k* + 1, and *T*_*loss*_(κ, *k*, …) to decrease the number of neighbors of a given neuron from *k to k* − 1 (see Figure 2 on top for a graph interpretation of the model). Assuming these depend on global topological aspects related with homeostatic considerations, such as the mean degree κ = 〈*k*〉 in the neural population, and local dependencies as the neuron degree *k*, this model explains the synaptic pruning curves observed in actual brains (Johnson et al., 2010; Millán et al., 2018). See the bottom left panel of Figure 2 for typical synaptic pruning profiles generated with this model. The functions *T*_*gain*_(κ, *k*, …) and *T*_*loss*_(κ, *k*, …) have a neurophysiological justification, as it is well known that neuron electrical activity regulates neural connectivity inducing axonal branch formation (Uesaka et al., 2006) and synaptic refinement (Vonhoff and Keshishian, 2017). Since neuron activity depends on the net current the neuron receives from its neighbors—being larger for increasing number of neighbors—ultimately it depends on its degree *k*. Also synaptic growth and death depends on the concentration of molecules that can diffuse through the whole neural medium, and given that calcium ions activate proteins involved in regulation of synaptic growth and pruning (Jourdain et al., 2003; Cornelia Koeberle et al., 2017), such processes cannot be considered only local. On the other hand, one may consider explicitly not only topological factors but also neurophysiological influences such as the synaptic currents arriving to each neuron *I*_*syn*_. This has allowed to investigate the interplay between brain function and network topology during development (Millán et al., 2018, 2019, 2021). More plausible realistic assumptions could be included within this framework, for example, the interplay between subcellular mechanisms such as intracellular calcium dynamics and astrocytes function since it has been recently reported that astrocytes actively contribute to synaptic pruning and developmental refinement of neural circuits in the brain (Lee et al., 2021).

## Looking into Our Minds

Another important aspect of the mind that is worth to be highlighted here—since it has practical and conceptual relevance, though one might at first glance feel it is just a technical aspect—is that transitions among mental states of qualitatively different properties often can be appropriately interpreted, using physics terminology, as *non-equilibrium phase transitions*. In fact, this concept (Marro and Dickman, 2005) in general concerns a transition among different macroscopic well-defined states (phases) in a system that, due to any fluxes or other interactions with the outside, cannot be in thermodynamic equilibrium, so that one cannot characterize in practice it by any Hamiltonian function. Concerning the brain, there are many facts that prevent equilibrium and from writing such a function, e.g., the different type of currents and fields that affect the neural excitability at the subcellular, cellular, and network level and time-dependent external fields such as the stimuli currents arriving from the senses. In spite of this and in analogy with a thermodynamic phase transition, the brain shows some relatively sharp changes at certain values of relevant parameters where its response to quite small perturbations exhibits a very large susceptibility and propagates in time and space without damping as in the familiar equilibrium criticality in, for instance, condensation. Under this particular condition, the brain is able to develop its characteristic cognitive functions such as decision tasks, attention breaks, optimal processing of information from the outside, or the processing of episodic like memories.

In other words, what we casually name “gray matter” is nothing but “condensed matter,” in the sense that it undergoes (possibly dynamic) changes formally similar to those shown by other materials, so much so that they are described with precisely the same mathematical structures (Marro and Dickman, 2005). That is, there are qualitative changes in the mind, whether they are dynamic while brain function or structural along the individual's evolution, that happen to be essentially equivalent in a formal sense (Marro and Torres, 2021) to *phase transitions* in physics, such as ferromagnetism, superconductivity, and superfluidity. The difference is conceptual more than practical in the sense that the later ones are of a thermodynamic nature, as they affect isolated systems in the state known as *thermodynamic equilibrium*. On the contrary, the mind and the nervous system are *open systems*, which experience stationary flows of matter, energy, and/or information with its surroundings and typically exhibit different types of inhomogeneity, so that they constantly are far from that equilibrium state. A practical consequence of the otherwise similarity between phases and mental states is that both, structure and dynamics of the brain are likely to be studied with very powerful methods and concepts developed in the study of matter and radiation (Marro and Dickman, 2005; Marro and Torres, 2021).

**Figure 3:**
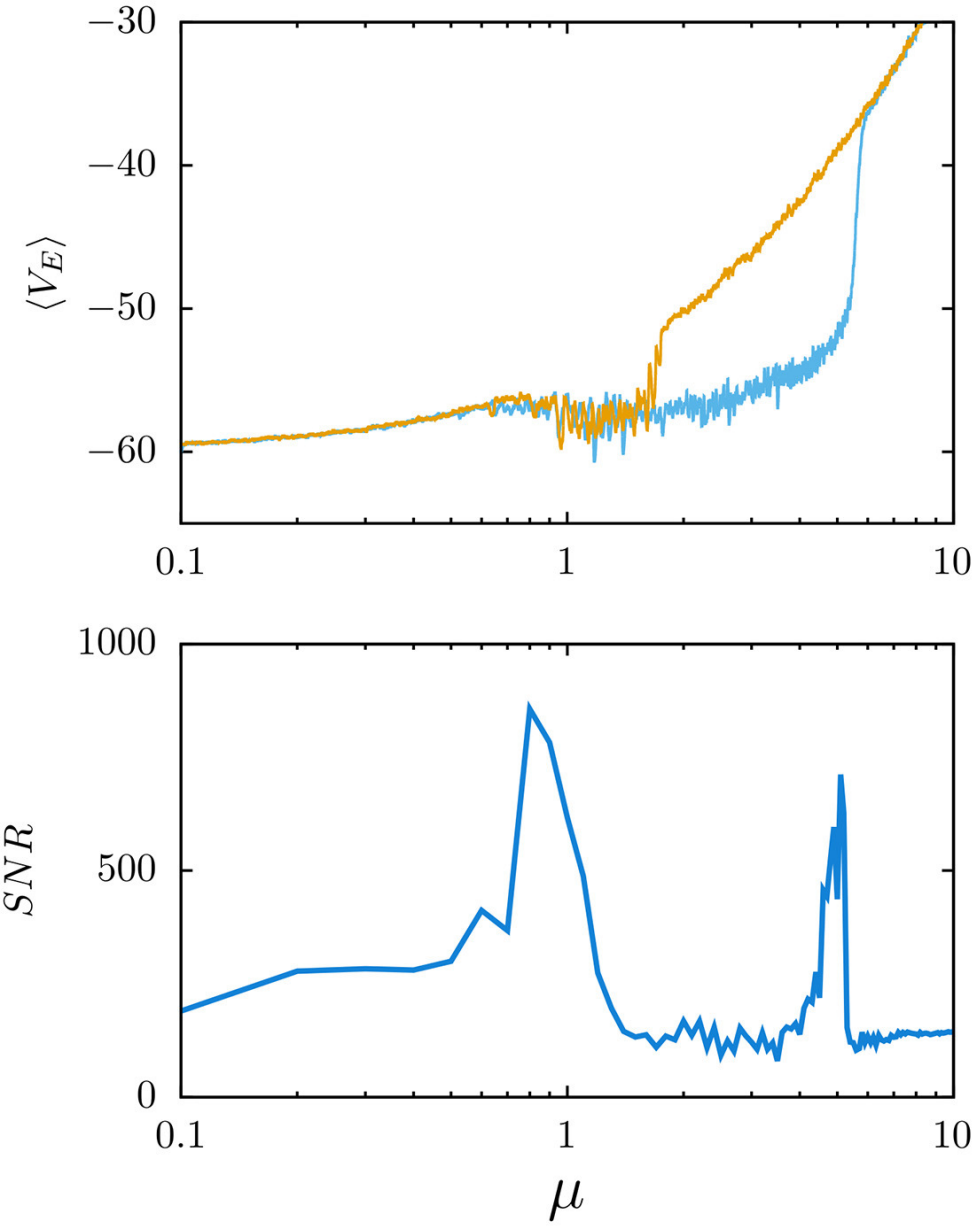
Electroencephalogram (EEG) waves in stochastic resonance phenomena. (Top) Model generation of waves, similar to the ones recorded in actual EEGs. This is for an excitation/inhibition balanced neural population, and the mean membrane voltage of all excitatory neurons is plotted as a function of the level of arriving excitatory uncorrelated inputs from outside of the population—measured by the parameter μ (the mean value of random depolarizing inputs that an excitatory neuron receives during 4 ms from other regions outside of the population)—and in the presence of short-term synaptic depression (Pretel et al., 2021). Note that the waves emerge around μ≈0.6 and disappear in an explosive phase transition for stronger noise levels (μ≈5). This is a consequence of depression, which reduces the effect of the strong activity of excitatory neurons over the inhibitory ones thus reducing the firing of inhibitory neurons and increasing further the activity of excitatory neurons. (Bottom) Signal-to-noise ratio (SNR) vs. μ when adding a weak sinusoidal input to each neuron while processing the weak signal, which allows detecting relevant phase transitions because of an enhancement of the SNR around the phase transition points.

This important fact happens to offer a solid, conceptual and mathematical, support thus washing out a certain mystery and consequent misgivings initially affecting to reports that the mind shows avalanches, seismicity, and critical and chaotic dynamics, which for a time was not considered befitting of the brain. Now it is quite clear why mental states often entail long-range correlations, as this occurs at critical points in physics where it is well known it allows any part of the system to be strongly receptive to what happens in any other, and vice versa. This is surely very important, since physics shows how the most extraordinary phenomenology emerges in this way. Moreover, the phenomena that are associated to such strong extensive correlations and large susceptibility in the mind, given the prevailing inhomogeneity and flows with the environment in this case, turn out to be even more varied and bizarre than in systems at thermodynamic equilibrium. Even more, by adding without reluctance the brain to the set of physical or, say, condensed matter systems, a new and exciting world becomes accessible to experiments. For example, it has been described how one may detect transitions between mental states by simple, e.g., “psychophysics” experiments that, studying the propagation of signals through the brain, report on the existence of *stochastic resonances* (Manjarrez et al., 2002, 2003; Yasuda et al., 2008; Torres et al., 2011). And it has thus been shown how these turn out to correspond precisely with *phase* transitions clearly denouncing very significant changes of the mind dynamics (Torres and Marro, 2015; Marro and Torres, 2021). Particularly, this has allowed to interpreting the celebrated cerebral “rhythms of activity”—those named *alpha, beta*, and *gamma waves* and *ultrafast oscillations*—whose existence was first revealed by the time series of EEGs long ago. Actually, a simple model has recently shown (Galadí et al., 2020; Pretel et al., 2021) how the changes between these types of oscillatory behavior are just transitions between *phases* or mental states that one can classify and decipher in neuroscience performing appropriate EEG and magnetoencephalography (MEG) experimental setups (see Figure 3).

**Figure 4:**
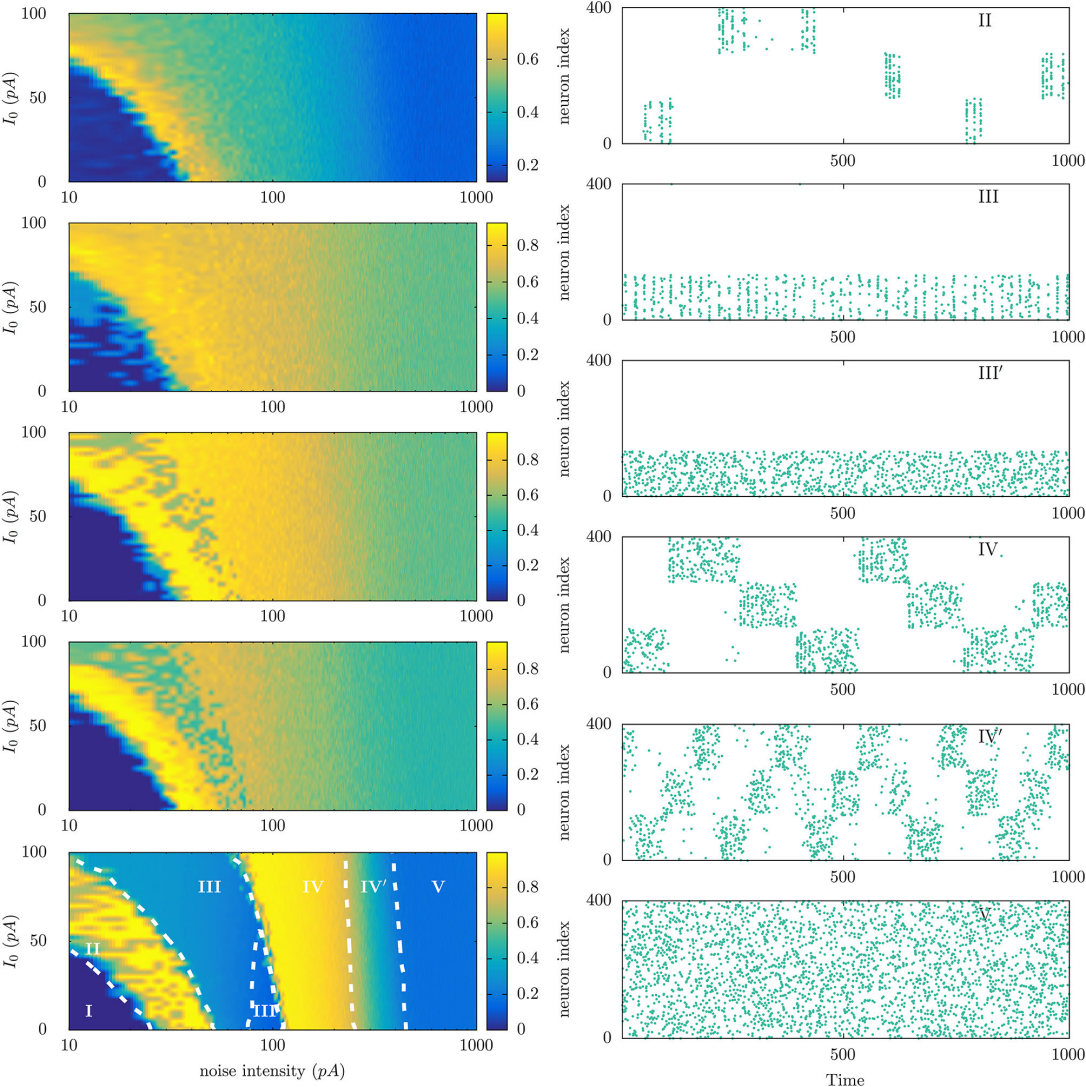
(Left) Phase diagrams showing the emergence of different non-equilibrium phases in neural populations that are connected with each other using different connectome data corresponding to different animal species. For each phase diagram, the vertical axis corresponds to the level of the constant current *I*_0_ injected to each neuron in the population to increase its excitability level, and the horizontal axis corresponds to the intensity of a noisy current also injected in each neuron to account for the possibility of random uncorrelated noises—arising from different sources—affecting neuron dynamics. Model details are described in Ref. Torres and Marro, 2015. From top to bottom connectomes are of *Caenorhabditis elegans* worm, pigeon, macaque, human, and a fully connected network. The color code represents the level of the time averaged overlap (ranged between 0 and 1) between the neuron population activity and any of the three particular patterns of activity stored at the synaptic maximal conductances. (Right) Raster plots illustrating the dynamical features of the different mental states observed in the parameter's regions marked with roman letters in the left bottom panel.

At the light of parallel situations in physics, one expects that a main detail within this scenario of emerging (non-equilibrium) mental phases will be the topology of the brain, which is certainly expected to condition its functions and interactions with the environment. In particular, one may anticipate that different species will exhibit a different chart of characteristic non-equilibrium mental phases, as illustrated in Figure 4 using a simple model implemented with a number of connectomes data (see Torres and Marro, 2015 for model details).

**Figure 5:**
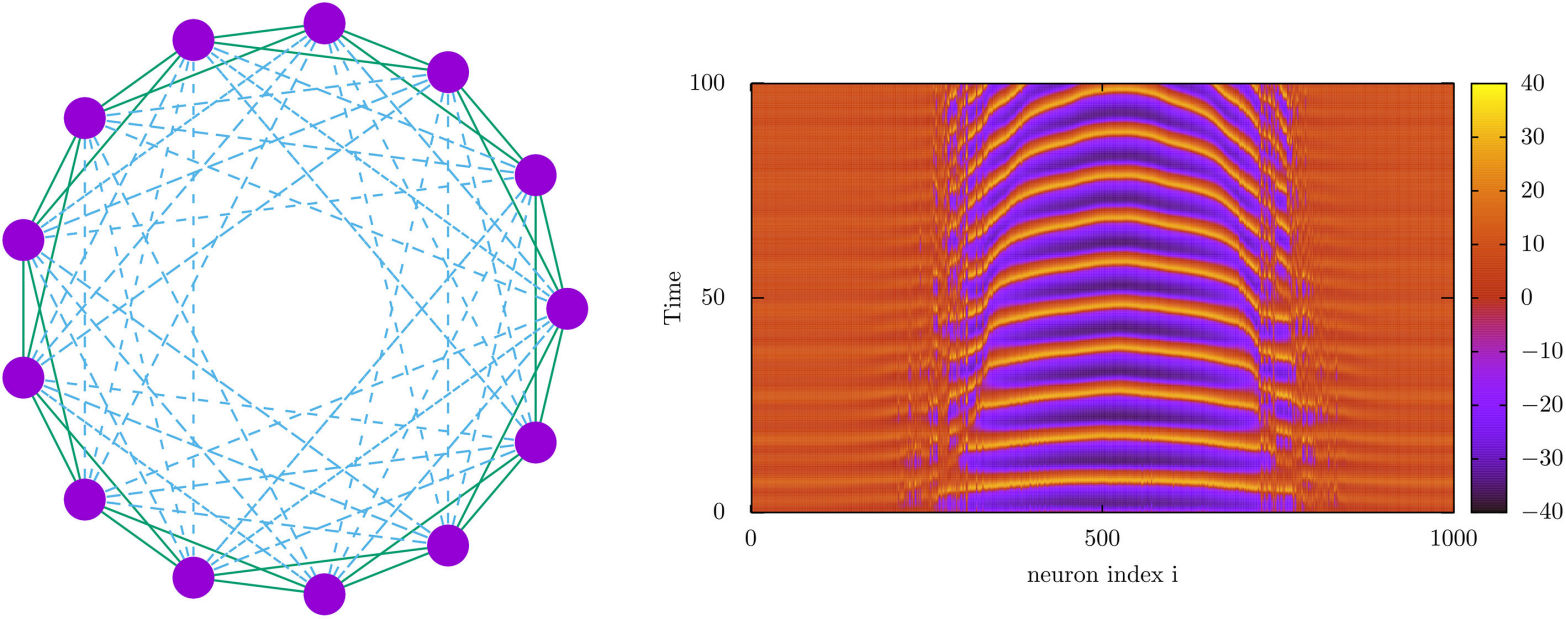
(Left) Network architecture used for the study of the emergence of *chimera* behavior using nearest neighbor electrical connections (solid lines) and long-range chemical synapses (dashed lines). (Right) Density plot depicting the time-dependent voltage traces of a 1,000 neuron population interconnected with the topology in the left panel (Calim et al., 2020). This shows the emergence of two chimeras separating different neuron subpopulations with different dynamical regimes, one (the center of the image) with high-amplitude normal spiking activity and the others (the non-centered regions) with high-voltage low amplitude oscillations, constituting a so-called *chaotic amplitude chimera* (Calim et al., 2020). Voltage membrane dynamics of the neurons (purple circles in the right panel) has been computed using a Morris–Lecar neuron model (Morris and Lecar, 1981).

Concerning mind phases, it was just shown (Calim et al., 2020) emergence of *chimera states*, namely, oscillatory phases where some neurons of the population are coherently oscillating in synchrony and the rest are oscillating out of phase in an asynchronous regime (see Figure 5). This seems robust in many situations, including spiking and bursting neuronal populations and complex network topologies including hybrid synaptic schemes with chemical and electrical connections. Therefore, they may possibly occur also in actual cases, where they might be related, for instance, with neural activity during uni-hemispheric sleep in dolphins and with the emergence of bumps of activity in working memory tasks (Compte et al., 2000). Since current studies have demonstrated their occurrence in parameter's regions between traveling wave phases and coherent synchronous phases, these states could be important to increase synchrony in some brain areas or to prevent epileptic seizures associated to traveling wave behavior.

## Discussion and Perspectives

Summing up, this perspective article presents some promising ideas and research lines for the study of the brain function based on simple biologically motivated neural population models that have been analyzed using statistical physics methods. This uncovers intriguing emerging phenomena due to cooperation of the systems basic elements, namely, neurons and synapses that are related by a complex network topology. In particular, we emphasize here the possibility that critical phenomena, similar to those in condensed matter, occur in the brain, which is quite sensible given the universality of the basic nature laws within such scenarios. We, thus, describe emerging phase transitions separating non-equilibrium phases that seem to characterize mental states as well as brain functions that seemingly involved by human identity and intelligence. No need to say that our (statistical physics) point of view here has limitations. For example, it is difficult to include all the microscopic details that can affect neuronal and synaptic dynamics, and some of them, including also the connection networks details, are yet to be fully described by neurobiologists. On the other hand, it yet needs to be clarified whether some brain functions are just emergent collective phenomena. For example, it is still difficult to fully understand and quantify how our subjective experience is coupled to the emergent process arising from the complex interrelation of the elements, mainly neurons and synapses. In any case, our approach here—also followed by many other colleagues—may be seen as a first meaningful analysis in which one may easily incorporate additional details as provided by new experiments.

On the other hand, it is remarkable how the whole of the framework above (see also Marro and Torres, 2021, and references therein) naturally leads to motivating extensions. In particular, returning to the notion of intelligence, it makes sense to assume within this scenario that, as a substantial part of the cognitive process, the mind constantly and quickly simulates relevant events and alternatives. That is, the mind would be producing, mostly unconsciously and for its own and immediate use, kind of well-informed “short films” including a variety of data, feelings and emotions pertinent to each case. This means that we instinctively imagine options that help us to decide at every moment what could be the most convenient in view of the “maximum” of available information (Wang, 2012). We can imagine that this maximum includes, in addition to all the data stored in our synapses relevant for the task in question, sensory data on the spatial and temporal environment and other predictions perhaps generated on the fly. In fact, an essential deficit in today's computers imitating intelligence would be this intimate relationship “back and forth” between memories and current processing that surely determines our decision-making and characterizes our brain functions.

Consequently, it seems that we may perceive *intelligence* as the result of all this, combined with the ability with which each individual is able to handle it. This is a human quality that shows to us as a kind of multifaceted device able of attending, perceiving, relating, and predicting. All this thanks to that critical, very effective dynamics described above commissioned to establish broad and rapid correlations between any part of our brain and the rest of our nervous system, including the senses as “windows” to the outside. In addition, our mind is so dynamic and adaptive that, not only does it generate new information using its warehouse to make predictions about situations that arise, but also reinforces or weakens our memories by adapting them to the success or failure of those predictions. Thus, apart from its relationship with the individual's ability to correlate, reason, resolve, etc., intelligence brings learning effectiveness. This increases our ability to anticipate threats and visualize even the most remote possibilities while reviewing the past, so that intelligence also seeks and, at best, achieves a better forecast of the future in our environment.

In this scenario, we may wonder about the control we have on our intelligence, besides circumstances that develop and activate (or not) throughout our lives under the rule of genetics, which probably determines, at least in part, aspects such as the intelligence capacity, disease propensity, or gender identity. In any case, it is not at all realistic to imagine that determinism governs the mind, e.g., a noise brain level is clearly noted during the state of consciousness (Lendner et al., 2020) and influence our behavior (Waschke et al., 2021). Moreover, the mechanisms involved are quite adaptive—meaning that they may be conditioned by eventual and rather random interactions with the outside, including social ones hanging on others—, and there are clear indications of defects and contradictions in relation to our decision-making processes. One source for these is surely the prejudices, traumas, and “manias” that, possibly hidden from ourselves, will induce biases not necessarily consistent with the reality in those “short films” we make. This, in addition to lessening our mind finesse and perhaps spoiling our best forecasts, would ruin a hypothetical determinism. Nor we should rule out that, as it is characteristic of natural phenomena, the aforementioned processes of memory, contrast, simulation, prediction, and decision include some randomness to its unconscious character. In short, decisions are probably made with certain autonomy, that is, without explicit real consent, that perhaps would only be explicitly communicated to us an instant after being taken without our will.

Endorsing the above, there is evidence that voluntary acts are preceded of subtle electrical changes in the brain, which would reflect a preparation process before the individual realizes it. In particular, perfecting experiments decades ago, recent EEGs and magnetic resonance studies show that the frontal cortex displays indications of the action to be performed a few seconds before the subject happens to “know” it (see, for instance, Soon et al., 2008; which confirms previous experiments of the neurologist Benjamin Libet in the 1980s), which suggests that the control we have of our own mind is limited. Actually, it seems that there are a few seconds in which we do not have a conscious supervision of each of our acts. This delay is compatible with the image of intelligence given above, according to which we evaluate—consciously, partially consciously or unconsciously—our options, which takes a finite time.

Also interesting are some consequences of the above on the concept of consciousness. Imagine that, at the request of an emergency phone operator, we have to distinguish whether a person just injured in an accident is “conscious or not.” Could we conclude with confidence? Back home, we check for consciousness in the thesaurus. It will say something like: “*our spontaneous knowledge, more or less vague and reflective, of the surrounding reality*.” OK but insufficient; it is important to note that it is not an eventual passive act, but a *perception*, where knowledge needs to be “perfected” with an intuitive element that constantly conditions us, to the point that it not only integrates us into the near context but also makes that we recognize ourselves in it. It follows that, as intelligence and identity, consciousness rests in memory, that is, in how we do to maintain huge stores of information and, quickly and automatically, we are able to recovering any specific portion that we might need. In short, these are human activities that require both interaction with the environment and ability to experience subjective sensations, so that they rest in the mind. According to what we have seen above, today one can say that consciousness and identity are a global property of the nervous system, especially in relation to the whole of its synapses.

We end up noticing that a main conclusion here may be that, as compared to other cooperative—natural or artificial—systems, an adequate activity of the intimate neural relationships is essential for the superiority of our minds. More than a century ago, when the matter was still believed to be a continuous medium, Santiago Ramón y Cajal noted the existence of those synapses that for him were “*mysterious butterflies of the soul whose beating of wings who knows if one day will clarify the secret of mental life*.” In a way, this is fully confirmed. We know that the versatility and power of the mind is inherent to the modulation on several time scales—kind of breathing, from calm to anxious—that these butterflies make of neuronal cooperation. They house indeed our intelligence, identity, and consciousness.

## Data Availability Statement

The raw data supporting the conclusions of this article will be made available by the authors, without undue reservation.

## Author Contributions

JT and JM contributed to conception and design of the study and wrote the manuscript. Both authors contributed to manuscript revision, read, and approved the submitted version.

## Conflict of Interest

The authors declare that the research was conducted in the absence of any commercial or financial relationships that could be construed as a potential conflict of interest.

## Publisher's Note

All claims expressed in this article are solely those of the authors and do not necessarily represent those of their affiliated organizations, or those of the publisher, the editors and the reviewers. Any product that may be evaluated in this article, or claim that may be made by its manufacturer, is not guaranteed or endorsed by the publisher.
